# A Randomized Controlled Trial Testing the Effects of a Social Needs Navigation Intervention on Health Outcomes and Healthcare Utilization among Medicaid Members with Type 2 Diabetes

**DOI:** 10.3390/ijerph21070936

**Published:** 2024-07-18

**Authors:** Amy McQueen, David von Nordheim, Charlene Caburnay, Linda Li, Cynthia Herrick, Lauren Grimes, Darrell Broussard, Rachel E. Smith, Dana Lawson, Yan Yan, Matthew Kreuter

**Affiliations:** 1 School of Medicine, Washington University in St. Louis, 660 S. Euclid Ave., St. Louis, MO 63110, USA; herrickc@wustl.edu (C.H.); yany@wustl.edu (Y.Y.); 2 Health Communication Research Lab, Brown School, Washington University in St. Louis, 1 Brookings Hall, St. Louis, MO 63130, USA; dvonnordheim@wustl.edu (D.v.N.); ccaburnay@wustl.edu (C.C.); lindali@wustl.edu (L.L.); l.grimes@wustl.edu (L.G.); mkreuter@wustl.edu (M.K.); 3 Louisiana Healthcare Connections, 4171 Essen Ln, 2nd floor, Baton Rouge, LA 70809, USA; darrell.broussard@cgifederal.com (D.B.); rachel.e.smith@louisianahealthconnect.com (R.E.S.); dana.lawson@louisianahealthconnect.com (D.L.); 4 CGI Federal, 538 Cajundome Boulevard, Lafayette, LA 70506, USA

**Keywords:** Medicaid, low-income population, chronic disease, social needs, intervention, randomized controlled trial

## Abstract

Health systems are increasingly assessing and addressing social needs with referrals to community resources. The objective of this randomized controlled trial was to randomize adult Medicaid members with type 2 diabetes to receive usual care (*n* = 239) or social needs navigation (*n* = 234) for 6 months and compare HbA1c (primary outcome), quality of life (secondary outcome), and other exploratory outcomes with *t*-tests and mixed-effects regression. Eligible participants had an HbA1c test in claims in the past 120 days and reported 1+ social needs. Data were collected from November 2019 to July 2023. Surveys were completed at baseline and at 3-, 6-, and 12-month follow-up. Health plan data included care management records and medical and pharmacy claims. The sample was from Louisiana, USA, M = 51.6 (SD = 9.5) years old, 76.1% female, 66.5% Black, 29.4% White, and 3.0% Hispanic. By design, more navigation (91.5%) vs. usual care (6.7%) participants had a care plan. Social needs persisted for both groups. No group differences in HbA1c tests and values were observed, though the large amount of missing HbA1c lab values reduced statistical power. No group differences were observed for other outcomes. Proactively eliciting and attempting to provide referrals and resources for social needs did not demonstrate significant health benefits or decrease healthcare utilization in this sample.

## 1. Introduction

Care management programs operated by health (insurance) plans assist members one-on-one by creating a personal care plan with follow-up calls to address barriers and access to interventions, including finding a doctor, making appointments, and ordering medical supplies or equipment covered by the plan. Care managers make phone calls daily to engage high-acuity patients, and standard practice is for care managers to focus on health needs. However, many care managers also attempt to address social needs reported by members, such as food, housing, and transportation, by providing mailed information (e.g., assistance application, nutrition education), referrals to community resources (e.g., food banks), and facilitating resources available through the health plan (e.g., transportation, free home glucose monitors).

Prior observational research has shown negative associations between social needs and health-related outcomes [1,2,3]. For example, having social needs has been linked to psychological stress, sleep disturbances, medication nonadherence, physical and mental health problems, hospitalization, and mortality [4,5,6,7,8]. Most social needs research in diabetic populations has focused on food insecurity, which has been associated with greater cost-related medication nonadherence, worse self-reported health, poor glycemic control, less fruit and vegetable intake, and lower diabetes self-efficacy [9,10]. Although the results specific to diabetes self-management and glucose control (HbA1c) are promising, many studies have involved small samples, rely on a physician’s referral, engage patients only in the short-term (i.e., 42 days), and are challenged by measurement, follow-up, and identifying appropriate comparison groups [2,11,12].

In 2017, when this study was proposed, most social needs intervention results were based on pilot studies. The results, especially for health outcomes, were mixed, and reviews of the literature identified multiple areas for methodological improvement [13,14,15]. In one review, intervention studies addressing housing needs found positive effects on health outcomes, costs, or both, whereas studies of nutrition, income, or care coordination supports were more sparse and had mixed effects [15]. An evaluation of Health Leads programs (not randomized trials) across three sites found that among patients who screened positive for at least one social need, 58% agreed to participate in needs navigation, and participants showed modest improvements over time in blood pressure and cholesterol but not in HbA1c compared with those who declined participation [16]. Reliance on health outcomes in electronic health records may ignore more proximal effects on patient-reported outcomes, including quality of life, depression, and stress.

The purpose of this report is to describe the results of a practical randomized controlled trial comparing the effects of a care management intervention that provided proactive social needs navigation to adult Medicaid members with type 2 diabetes vs. usual care. Research questions included the following: (1) Whether navigation vs. usual care participants would have much greater engagement (dose) in care management, especially for social vs. health needs. (2) Whether participants would evaluate navigators positively, even though none requested care management services. (3) Whether groups would differ on HbA1c test values (primary outcome) and quality of life (secondary outcome) at 6 months follow-up. To inform future studies and meta-analyses, we explored group differences in additional health, healthcare utilization, and psychosocial outcomes using available data at 6 and 12 months follow-up.

## 2. Materials and Methods

### 2.1. Population and Study Setting

Adult (age 18+) members of the state-wide Medicaid health plan, Louisiana Healthcare Connections, were recruited November 2019–July 2022. Final data collection was completed by 5 July 2023. Claims data were used to identify members with type 2 diabetes (defined as ≥1 inpatient or ≥2 outpatient medical claims with types 2 diabetes relevant ICD-10 codes) and a recent (<120 days) HbA1c test for study recruitment. Identified members were excluded from recruitment if they were involved in care management in the past 90 days or if they did not have a phone number or independent address (e.g., no rehab, nursing home, or correction facilities).

### 2.2. Recruitment and Randomization

The health plan shared contact information of identified members with research staff who attempted to contact them by phone to introduce the study, invite participation, obtain verbal informed consent, conduct additional eligibility screening, and administer the baseline survey. To be eligible for the study, members could not be pregnant or only have a history of gestational diabetes, could not have cognitive or hearing impairments that impede research participation by phone, could not report that their latest A1c was <7.0, and had to report having at least one unmet social need (see measures). All recruitment calls and materials were in English. Participants who completed the baseline survey were randomized in equal proportions using a predetermined list of random numbers in REDCap by research staff to one of two study conditions: usual care (survey only) or proactive social needs navigation. Staff manually randomized participants, and their assignment was documented in REDCap after the baseline survey was completed. Group assignment was not blinded to team members with relevant REDCap permissions, and identifiers of those assigned to navigation were sent to the navigator. Participants were sent a USD 50 gift card for completing the baseline survey. Participants received a USD 25 gift card for each follow-up phone survey completed at 3, 6, and 12 months post-baseline. All surveys were administered by phone by trained research assistants who recorded participant responses in real-time using REDCap survey forms.

### 2.3. Alterations to Original Study Protocols

Due to the COVID-19 pandemic, the study team was not allowed to communicate with Medicaid beneficiaries between 13 March and 13 July 2020. The deadlines were extended for completing each follow-up survey by 3 months to account for this pause in communication and applied the cutoffs to all participants. The 82 participants recruited before the recruitment pause were less likely to complete later follow-up surveys compared to participants recruited after (at 6 months; pre-pause: 29.3%, post-pause: 77.0%, *p* < 0.001; and at 12 months; pre-pause: 50%, post-pause: 69.3%, *p* = 0.001).

### 2.4. Social Needs Navigation Intervention

Usual care participants could receive care management services if identified and referred by the health plan or providers to address high–acuity needs. The focus of the Social Needs Navigation intervention was to proactively help participants not otherwise identified for care management to address unmet social needs. If asked, navigators also offered encouragement, information, and resources related to health needs, but this was not their primary focus for outreach calls. On the first call, navigators introduced themselves, established rapport, and explained their role in identifying helpful resources. Rapport-building typically involved asking members an open-ended question to ascertain personal interests, hobbies, and activities enjoyed as a way of using related examples in discussions, but such techniques and results were not formally tracked or analyzed. Navigation continued for up to 6 months post-baseline. Even if participants were not actively working on addressing an unmet need, the navigator would follow-up periodically to assess any changes. The number and frequency of calls were determined by participants’ needs, interests, and willingness to interact. Either party could initiate a call, but on every call, navigators were expected to (1) review unmet needs previously reported and ask participants to report any other needs; (2) review progress made in addressing the need; (3) identify possible resources; (4) evaluate eligibility for resources and/or barriers to achieving goals; and (5) provide referrals (by phone and mail). In cases where navigators were aware of the gaps in needed services or long waitlists (e.g., permanent housing), they discussed priorities and timelines with participants and sought solutions in other need categories with more available resources (e.g., food, utilities), which also may free up money for the participants’ primary unmet need. Navigators provided instrumental support (e.g., providing reminders, sending eligibility forms, arranging transportation) and social support through regular contact, attempting to improve stability and security for participants longer-term.

### 2.5. Navigator Background, Training, and Supervision

Two navigators (one social worker and one with comparable work experience), hired by the health plan, sequentially delivered the intervention. Both possessed outstanding interpersonal communication skills and experience working with low-income populations, including their use of rapport-building techniques, which are common in case management outreach. Both received additional training from the research team in basic research methods, social needs identification and problem-solving, and counseling approaches. Navigators were familiar with many resources available through the health plan and state and local communities in which members lived and often sought out new resources. Navigators used the health plan’s care management database (TruCare) to track navigation interactions and progress over time for each participant. The second navigator trained with the first navigator for over a month before the position was transferred in November 2011 to ensure consistency. Also, during the intervention period to maintain fidelity, each navigator received individualized supervision and feedback weekly from research team members with regular opportunities to ask questions, discuss challenging cases, and brainstorm solutions. Navigators also received standard evaluation and oversight as care management staff (e.g., HIPAA protections, customer service quality). Although personal characteristics may have created subtle differences, the standardization of the outreach approach and content of discussions and referrals likely increased the similarity of participants’ experiences with the intervention.

### 2.6. Data Sources and Measures

#### 2.6.1. Medical Claims

Medical claims were aggregated for the year following each participant’s enrollment in the study. Claims are collected for the purpose of reimbursement and differ from clinical care data recorded by providers in patients’ medical records. As it was beyond the scope of this study, the research team did not validate medical claims against patient medical record data. ICD-10 codes were used to identify diagnoses, whereas CPT or procedure codes were used to identify outpatient procedures or services.

HbA1c test dates were available in claims, and the health plan attempted to obtain lab values for the baseline and the first test completed 6–12 months post-baseline for each participant to assess change over time (primary outcome). Many requests to providers and labs to share HbA1c test results went unfulfilled, so analyses of HbA1c values are based on a subset of participants, but not equal to the required number for appropriate statistical power. The amount of missing data precluded the use of multiple imputation methods in analysis and instead produced hypothesis-generating information rather than hypothesis-testing results [17].

Additional exploratory measures were examined with claims data, including recommended health screenings: eye exams, kidney function panel, and lipid panel (see codes in Supplementary Table S1 from HEDIS Diabetes Care Guidelines, 2022–2023 [18]).

Diabetes complications variables included uncontrolled diabetes, which was defined as any claim with a diagnosis code for hypoglycemia (ICD-10: E10.64 or E11.64) or hyperglycemia (ICD-10: E10.65 or E11.65) [19]. Place of service and procedural codes were used to distinguish between uncontrolled diabetes presenting in an outpatient or emergency department/inpatient setting. The Diabetes Complications Severity Index (DCSI) [20] was used to provide comparison with a recent observational study investigating social needs in a publicly insured population with diabetes [3]. The DCSI uses ICD-10 diagnosis codes to calculate scores for 7 categories of diabetes complications, which range from 0 to 2, depending on the severity of the diagnosis codes present, except for neuropathy, which only ranges from 0 to 1. Sum scores range from 0 to 13; higher scores have been shown to predict elevated risks of hospitalization and mortality [20,21].

Medication adherence variables were created from pharmacy claims data, including a count of unique prescription medications, and adherence to two medications commonly recommended in diabetes management: oral hypoglycemic medications used to manage blood sugars (e.g., sulfonylureas, meglitinides, biguanides; not insulin) and statins used to manage hypercholesterolemia (e.g., atorvastatin, rosuvastatin, simvastatin). Adherence to either drug class was dichotomized as having refills that covered 80% or more of the follow-up period (yes/no) for participants prescribed those medications [3].

Healthcare utilization variables were created from medical claims data. Wellness visits (none, any) were calculated from claims for preventive medicine services with CPT codes 99381–99397. Emergency department (ED) utilization was calculated by counting the number of unique ED service claim dates. ED utilization was also dichotomized at ≤4 visits vs. >4 to compare lower vs. higher utilizers. Hospitalization was calculated by counting the number of unique visits based on a managed inpatient calendar linked to inpatient authorizations and claims data. Although the identification of inpatient visits may include both planned and unplanned hospitalizations, those related to pregnancy and childbirth (*n* = 1) were specifically excluded. For people with an inpatient visit, rehospitalization within 30 days (none, any) was coded by comparing the unique admission dates determined for hospitalization.

#### 2.6.2. Navigation Records in TruCare Care Management Software Program

Process evaluation for receipt of intervention, as assigned, was determined using navigation records. Care plans are created after the care manager/navigator makes contact with a member and establishes the priorities for care management/social needs navigation. Care plans are only created for members who want to engage with the care manager/navigator and work together to address a health or social need. The care plan file links to sub-files that contain text descriptions of global problems (e.g., food, utilities, disability services, medication issues, home repairs), goals (e.g., obtain safe affordable housing, obtain money for car repairs, apply for utility assistance program, lose weight, obtain appointment with new primary care doctor), interventions (e.g., navigator mailed information, application form, called doctor, discussed options), and barriers (e.g., lack of community resources, depression, lack of motivation to change, low health literacy, limited mobility), among other written “notes”. In general practice, files are typically closed within 90 days by care managers, but for intervention participants, navigators maintained interactions for 6 months.

#### 2.6.3. Survey Measures

The Short-Form Health Survey (SF-12v2) was used to measure health-related quality of life (secondary outcome) at baseline and 6 and 12 months follow-up. Aggregate and subscale scores were created for physical health (physical role and functioning, general health, bodily pain) and mental health (emotional role, social functioning, vitality, and general mental health) using proprietary scoring algorithms [22,23]. The SF-12 has been shown to produce similar results in longitudinal studies compared to the longer SF-36 measure, so it was selected to reduce participant burden [24]. Also, the literature provides evidence of its validity for people with diabetes [25]. A change of 5 points in the aggregate scores reflects a moderate effect size with significant influences on healthcare utilization and expenditure [26].

Additional survey measures were used to describe sample characteristics, intervention satisfaction, and to explore additional differences by study group.

Socio-demographic and health factors, such as age, sex, race/ethnicity, education, income, and marital status, were assessed with standard items at baseline only. Single items assessed having a regular doctor, diabetes medications (oral agents, insulin), and most recent HbA1c value. A count of chronic conditions was created from a list of 21 [27,28].

Social Needs were assessed at all time points. Ten items assessed the likelihood that each participant’s personal safety, housing, food, transportation, child care (if applicable), social interaction, and various financial needs would be met in the next month [29,30]. Most response options ranged from 1 = very unlikely to 4 = very likely. One item that measured neighborhood safety and response options ranged from 1 = very safe to 4 = very unsafe. One item assessing space in the home included three response options: not enough space, about the right amount, too much space. To create dichotomous variables, needs were considered met when they were very unlikely or unlikely to arise in the next month; all other responses identified unmet needs. The total amount of unmet needs was a sum of the 12 dichotomous responses. Per the eligibility criteria, all the participants were required to report at least one unmet need at baseline, although scores of 0 were possible at follow-up.

Satisfaction with navigators was assessed using items adapted from national survey measures of patient satisfaction. Participants were asked to rate the extent they agreed (1 = strongly disagree to 4 = strongly agree) that study navigators were easy to talk to, helpful, trustworthy, cared about them as a person, were expert at helping them solve problems, and understood their life situation. Participants also indicated whether the number of calls was too much, about right, or not enough, and whether they would recommend the navigator (yes/no).

Psychosocial measures assessed at all 4 survey time points included Cohen’s 4-item perceived stress scale (α = 0.56) [31], a 2-item diabetes distress scale [32,33], the Patient Health Questionnaire-2 (PHQ2) depression screener [34], an 8-item scale assessing self-efficacy for performing diabetes self-care behaviors (α = 0.82) [35], an adapted 4-item social support measure (α = 0.86) [36], and 4 items adapted from the Pittsburgh Sleep Quality Index [37,38]. Diabetes self-management was measured by the 10-item Summary of Diabetes Self-Care Activities scale assessing the frequency of following recommendations for healthy diet, exercise, blood glucose testing, and foot care in the past 7 days [39]. Scores reflect the mean number of days participants performed each behavior.

### 2.7. Data Acquisition and Analysis

The research team worked closely with the health plan to obtain all the necessary approvals for secure access to limited datasets. The study was approved by the Institutional Review Boards for Washington University and Louisiana Department of Health and Human Services for all study materials and procedures.

**Figure 1:**
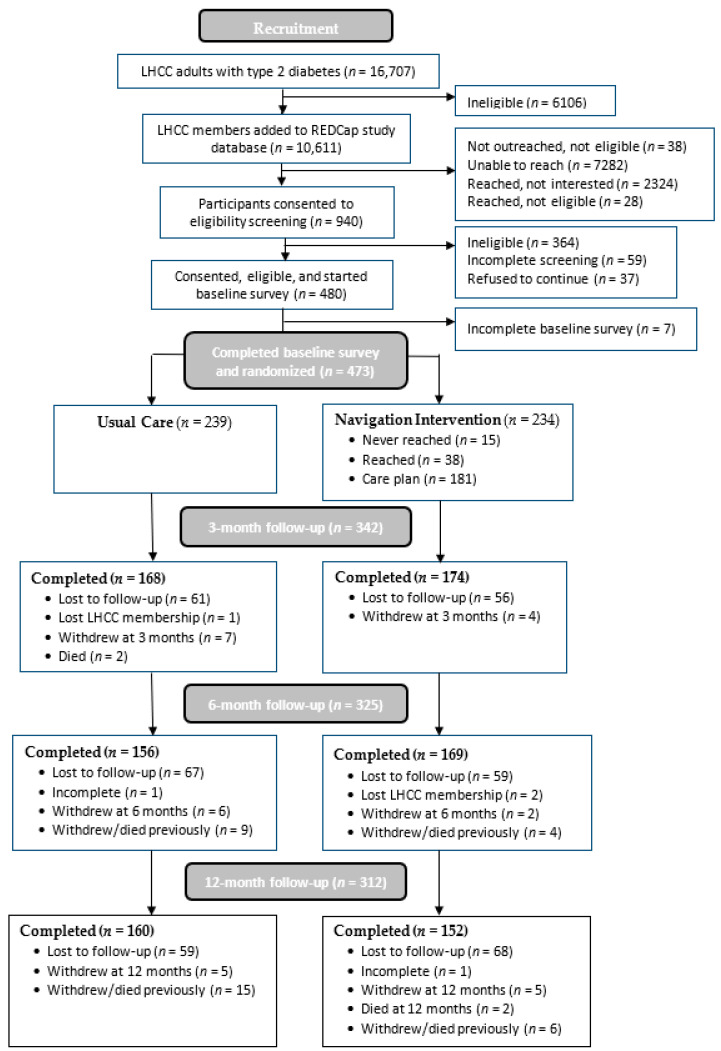
CONSORT flowchart of participant recruitment and retention.

Following CONSORT criteria for intervention trials, participant enrollment, eligibility, and retention rates are reported in Figure 1. Group differences in study outcomes were compared with *t*-test analysis. For quality of life, linear mixed-effect regression analysis was also used to evaluate group–time interactions to determine whether any changes over the three survey time points (time coded: 1–3) differed between the control and navigation groups. Mixed-effect regression analyses do not use listwise deletion but rather use all the available data from participants. These models produce unbiased estimates assuming data are missing at random. Missing at random assumes that the missingness depends on the observed data only, which is a common assumption for multiple imputation. Research suggests that mixed models are effective at addressing missingness in repeated measures data, and this approach is recommended over multiple imputation [40]. Additional exploratory analyses using other outcomes from claims and survey responses were conducted using chi-squared analyses, z-test for count data, and *t*-tests, and repeated survey measures were also analyzed with mixed-effect regression. Exploratory analyses can inform the design of future studies and meta-analyses, although the number of comparisons can inflate type I errors, so a method for controlling the false discovery rate was used. For example, a Bonferroni correction for the group comparisons of survey measures would interpret statistical significance as 0.05/38 = 0.0013, whereas the Benjamini and Hochberg method is more powerful and ranks observed p-values before comparing against a fraction of 0.05 [41]. The results of unadjusted analyses are reported due to the use of randomization and no evidence of study group differences in attrition. All analyses involving health plan data were completed using a virtual desktop interface with SAS version 9.4 (SAS Institute Inc., Cary, NC, USA), and R v4.2.3 (R Core Team, Vienna, Austria). Survey data were collected and retained on university servers and analyzed using SPSS (IBM Corp., Armonk, NY, USA) and R v4.2.3.

### 2.8. A Priori Power and Sample Size Estimation

With a total sample of 265 participants with type 2 diabetes, we would have 90% power to detect a 0.4% [42,43] mean difference (SD = 2) in HbA1c between the intervention and usual care groups using a two-tailed paired *t*-test with alpha = 0.05. A change in HbA1c = 0.5% is considered clinically meaningful [44,45], and every 1% reduction is associated with significant risk reduction [46,47]. To compensate for an estimated 30% loss by 12 months follow-up due to participant refusal or loss of Medicaid coverage, and the unknown pattern of HbA1c testing (and missingness) in our sample, we proposed to enroll 500 participants (*n* = 250 in each group). With *n* = 500 and a two-tailed chi-square test, we would also have >80% power to detect a 10% difference between groups among those who reduced HbA1c by 0.5% when the proportions of each study group are ≤25%. With 30% loss (*n* = 350), we would have over 0.90 power to detect mean group differences of ≥3.0 (SD = 12) in QOL scores at 6 or 12 months follow-up.

## 3. Results

The study involved 473 participants who completed the baseline survey and were randomized and formed the intent-to-treat sample (Figure 1). No differences were found in attrition by study group for withdrawal rates or follow-up surveys completed). Baseline characteristics are reported in Table 1. The sample was mean age 51.6 (SD = 9.5), 76.1% female (*n* = 359), 29.4% White (*n* = 137), 66.5% Black (*n* = 310), 3.0% Hispanic (*n* = 14). Most participants had Medicaid through either expansion (*n* = 250, 53.0%) or SSI (*n* = 189, 40.0%).

The first objective was to assess whether all 234 participants assigned to the social needs navigation intervention engaged in care management more than those assigned to usual care (dose). Notes from study navigators confirmed that 17 participants assigned to navigation did not respond to multiple outreach attempts; another 3 either did not engage until months into the intervention period or did not report any unmet needs or navigation goals. Thus, only 214 (91.5%) participants assigned to the navigation group had a care plan opened by a navigator (Supplementary Table S2). In contrast, 16 participants in usual care (6.7%) had a care plan opened by a care manager.

Although not qualitatively analyzed, in reviewing example notes from navigators, many calls with participants addressed food insecurity (e.g., participants were offered a referral to a food bank) or assistance paying utilities (e.g., participants were offered a referral for the Low-Income Home Energy Assistance Program [LIHEAP]). Many participants also identified health-related needs, such as referrals for a new primary care provider, dental care, or assistance acquiring medical equipment (e.g., glucometer, wheelchair, blood pressure cuff). Although all participants were required to identify at least one unmet basic need at the time of their enrollment, many told the navigator that this need had been resolved or they did not have the need currently. These observations illustrate the natural variability in social needs and changes over time.

Of those in the navigation group who completed a three- or six-month follow-up survey, only slightly more than half (57.4%) reported that they talked with a navigator. Of those that responded affirmatively, most evaluated the navigators positively, e.g., easy to talk to, trustworthy, cared about me, understood my life situation (mean scores > 3.5). Nearly all who responded thought that the total calls received was about the right number (92.9%) and would recommend the navigator to others (96.1%).

Few differences were observed by study group on claims data outcomes between baseline and 12 months follow-up (Table 2), and similar results were found when limiting analyses to the 6-month intervention period only. A priori plans for analyzing HbA1c values were limited by missing data, and no statistically significant benefits of navigation were observed. Despite nearly complete claims data for participants, small differences between groups produced few statistically significant findings in exploratory outcomes. Average DCSI scores were significantly higher for the navigation group vs. the control group, both in the year prior to baseline [usual care: M = 1.53 (SD = 1.64), navigation: M = 2.11 (SD = 1.94); η^2^ = 0.03, *p* < 0.001] and in the year after baseline [usual care: M = 1.70 (SD = 1.78), navigation: M = 2.15 (SD = 1.99); η^2^ = 0.02, *p* = 0.011], although the between-group differences were small. DCSI scores also increased significantly for both groups in the year after baseline compared to the year prior, although the effect was small (*r^2^* < 0.01, *p* < 0.05). There was no significant time–group interaction on DCSI scores, such that the rate of increase from the year prior to baseline to the year following did not vary between study groups.

Although the navigation group reported more anticipated needs for paying for utilities compared with the usual care group, after adjusting for the number of comparisons, no differences by study group were found for quality of life or other (exploratory) outcomes using survey data at 12 months follow-up (Table 3). Similar results were found at the end of the intervention period at 6 months follow-up (Supplementary Table S3).

Of all comparisons using all four time points of the survey data, only two significant group–time interactions were observed. Both were from subscale measures (SF-12 social functioning) and glucose monitoring, favoring the usual care condition, and would not be statistically significant after adjusting for the number of analyses conducted (Supplementary Table S4).

## 4. Discussion

Proactive outreach to members not already selected for care management was successful, and navigators reported that the engagement rate was much higher than in standard practice, possibly because members had already been successfully reached and enrolled in a longitudinal research study before navigators made outreach calls. Also, all participants had to report at least one unmet need to be eligible for the study; thus, most participants could directly benefit from talking to a social needs navigator. Although navigators documented interventions and goals completed, many global problems persisted. Some problems, such as food insecurity, have more plentiful options for resolution than others (e.g., housing, dental providers, car repair) [48]. Gaps in those who receive assistance from social needs interventions, differences by the type of assistance or hand-off received, and the availability (long-term) for resources to be received have been noted in the literature, along with calls for better collaboration between health and social service organizations [49,50,51,52].

Despite reaching most members assigned to navigation, over a third of those did not recall it. Providing navigator’s names may have improved recognition. To remedy the effect of remote interaction, navigators sent each participant a letter with their name, contact information, photo, brief description of their professional background, and introduction to their role as a navigator. In a qualitative study of Medicaid members in another state, the results showed that half of the participants invited to participate in a case management program did not recall being called, and some members who had participated in case management services over time did not accurately recall participating [53]. Brief introductions by phone and multiple contacts between members and various healthcare personnel may not be as memorable as intended by health plans and research studies. There may be advantages to engagement if patients perceive all healthcare personnel to be working together and sharing roles, especially when care managers can help obtain prescriptions for medications or supplies, arrange transportation to appointments, and send nutrition and physical activity resources by mail. However, the evaluation of specific services or individuals may be difficult in this situation.

The number of programs addressing social needs at the point-of-care in the US is increasing, but a minority have examined effects on health [54,55,56]. Few have reported positive effects on health [12], some have reported null effects [57,58,59,60,61], and some have showed increased utilization [62,63]. Similar to our study, recent reports found no effect on HbA1c [12,60,61] or quality of life [60]. Future reviews should examine which intervention approaches are most effective for addressing single vs. multiple types of needs, and how they may affect different health outcomes.

Reviews of prior social needs interventions have noted the large variability, so future studies will need to examine mechanisms of social needs resolution and effects on health outcomes and healthcare utilization. There is no consensus on whom to target for assistance, how often needs should be assessed, which needs should be prioritized, and how long to provide intervention. In this study, members who were not already identified for care management were eligible for recruitment with the goal of preventing insecurity and crises that lead to greater healthcare utilization and poor health outcomes. Although focusing on higher-acuity members may prompt “regression to the mean” in longitudinal studies, there may be greater return on investment for addressing social needs in this subgroup. Also, in-person navigation may be more engaging than remote or less personalized connections [61,64]. Most studies that engage patients at the point-of-care to assess and address social needs are (at least initially) in-person, which may prompt more positive perceptions if navigators are associated with patients’ healthcare provider. Such programs, however, often engage far fewer of the patients who report any needs [54,65]. Interventions that provide referrals to community resources may be most effective for people who have not been previously connected or who are more open to such an interaction, which may involve lengthy eligibility assessments that further burden the individual with more demands on their limited financial and cognitive resources. Future studies should go beyond asking patients to rate the appropriateness and feasibility of assessing social needs in healthcare systems to examine the perceived burden, stigma, frustration, and fatalistic views that may emerge, or examine the competing demands that are increased, if participants’ social needs are not easily met. Addressing social needs through community resources may not immediately impact physical and mental health outcomes at a group level, but longer-term benefits may be accrued if patients feel supported, gain self-confidence in finding resources, or improve collaboration with their healthcare providers. Also, future studies may find greater effects with longer intervention and follow-up intervals. Stepped interventions with increasing intensity or personalization of intervention approaches may be more effective, efficient, and sustainable. Greater examination of effects on mediating variables is needed; although, in this study, no group differences were observed for psychosocial outcomes such as depression, diabetes distress, and diabetes self-management self-efficacy.

## 5. Limitations

This sample of adult Medicaid members with type 2 diabetes was from a large but single health plan in Louisiana, and participants had to report an unmet social need to be eligible for the study; thus, they may not represent the full Medicaid population. Other differences in demographics and health conditions of Medicaid populations exist across states due to differences in program eligibility. Additionally, this study excluded people who were not living independently (e.g., rehabilitation facility) due to differences in how social needs would be reported and addressed. Ethically, it may seem problematic to exclude such individuals from a social needs intervention; however, this exclusion was only used at enrollment, and navigation outreach may not be a benign intervention if recipients are in acute crisis or unable to make independent decisions about their living situation. Their inclusion at baseline may also have delayed and reduced the likelihood of creating care plans and resolving social needs. Future studies may benefit from stratified randomization or targeted interventions by level of acuity or intervention setting differences. Study recruitment and intervention was halted during the early months of the COVID-19 pandemic, causing an interrupted timeline and intervention for the first 82 participants enrolled and lower response rates after the COVID-19 pause. As in all research studies with opt-in enrollment, study participants likely represent a biased sample of all eligible individuals. We lessened the impact of selection bias with multiple recruitment attempts by phone, mail, and/or email over time of all eligible members and through randomization to study group. A strength of this study is the use of both self-report survey data and medical claims data over 12 months, although each have limitations, including under-reporting. The intervention was conducted in real-world conditions by care management staff by telephone and mail only. Reaching participants by phone for recruitment and the first navigation call were slower after the COVID-19 pause and for the second navigator, respectively. Future studies may benefit from the ability to send two-way text messages. Other interventions have recruited from patients at the point-of-care, whereas this study recruited health plan members state-wide. The health plan was not able to obtain many HbA1c lab values associated with claims for testing, which limited power for detecting differences in this primary health outcome. Caution in interpreting these results is necessary because the data can neither support nor reject study hypotheses about group differences in HbA1c values. In contrast, few participants were missing claims data, but no significant group differences in utilization or other outcomes were found.

## 6. Conclusions

This practical trial capitalized on existing care management infrastructure for delivering a proactive social-needs-focused intervention to adult Medicaid health plan members with type 2 diabetes that reported at least one unmet social need who were randomized and followed for 12 months using survey and claims data. Although there is growing interest in routine assessment and referral for social needs, this study found no difference in health outcomes and utilization by study group. Additionally, no group differences were observed in potential mediating variables such as diabetes self-management behaviors, stress, and social support. Greater effects may be observed for higher-acuity samples and more intensive or in-person navigation. Small group differences at the population level with millions of Medicaid members in the US may be statistically significant, but future research is needed to affirm the clinical- and cost-effectiveness of intensive, proactive social needs navigation.

## Figures and Tables

**Table 1:** Sample characteristics self-reported on baseline survey (*n* = 473).

		Usual Care (*n* = 239)	Navigation (*n* = 234)
		*n* (%) or M (SD)	*n* (%) or M (SD)
Socio-demographic and health factors			
Age		51.31 (9.71)	51.79 (9.35)
Sex	Male	59 (24.7%)	54 (23.1%)
	Female	180 (75.3%)	179 (76.5%)
Race/Ethnicity	Non-Hispanic White	66 (27.6%)	65 (27.8%)
	Non-Hispanic Black/African American	152 (63.6%)	150 (64.1%)
	Other ^a^	17 (7.1%)	15 (6.4%)
Education	<High School	74 (31.0%)	77 (32.9%)
	High School/GED	77 (32.2%)	74 (31.6%)
	Some college/degree	88 (36.8%)	81 (34.6%)
Annual income	<USD 10,000	131 (54.8%)	125 (53.4%)
	USD 10,000–<20,000	52 (21.8%)	62 (26.5%)
	>20,000	37 (15.5%)	34 (14.5%)
Marital status	Married/living with partner	56 (23.4%)	51 (21.8%)
	Other	183 (76.6%)	182 (77.8%)
Has a regular doctor	No	6 (2.5%)	4 (1.7%)
	Yes	233 (97.5%)	229 (97.9%)
Sum comorbid conditions		7.61 (3.07)	7.56 (2.99)
Oral antidiabetic use	No	18 (7.5%)	13 (5.6%)
	Yes	221 (92.5%)	220 (94.0%)
Insulin use	No	106 (44.4%)	79 (33.8%)
	Yes	133 (55.6%)	154 (65.8%)
Most recent HbA1c value	7.0% to 7.4%	38 (15.9%)	32 (13.7%)
	7.5% to 7.9%	14 (5.9%)	12 (5.1%)
	8.0% to 8.4%	28 (11.7%)	25 (10.7%)
	8.5% to 8.9%	8 (3.4%)	10 (4.3%)
	9.0% or higher	68 (28.5%)	68 (29.1%)
	Unsure	83 (34.7%)	87 (37.2%)
Social Needs	Sum score	2.85 (1.67)	2.94 (1.77)
	Food	33 (13.8%)	36 (15.4%)
	Transportation	45 (18.8%)	36 (15.4%)
	Place to stay	13 (5.4%)	11 (4.7%)
	Living space	52 (21.8%)	43 (18.4%)
	Neighborhood safety	34 (14.2%)	35 (15.0%)
	Utilities	88 (36.8%)	82 (35.0%)
	Necessities	92 (38.5%)	88 (37.6%)
	Unexpected expenses	186 (77.8%)	185 (79.1%)
	Social isolation	59 (24.7%)	67 (28.6%)
	Personal harm	19 (7.9%)	23 (9.8%)
	Childcare	1 (0.4%)	9 (3.8%)
	Other	59 (24.7%)	73 (31.2%)

^a^ Other race/ethnicity: Asian American (*n* = 3), American Indian/Alaska Native (*n* = 7), Hispanic/Latino (*n* = 14), other/multiple race (*n* = 8). Legend. *n* = sample size, M = mean, SD = standard deviation, GED = General Education Diploma, HbA1c = glycated hemoglobin. No group differences were found. Statistical comparisons were not made when cell sizes *n* < 5.

**Table 2:** Healthcare claims outcomes from baseline to 12 months follow-up by study group (*n* = 473).

	Usual Care (*n* = 239)	Navigation (*n* = 234)	
Claims data:	*n* (%) or M (SD)	*n* (%) or M (SD)	Group difference
No medical claims post-baseline	2 (0.8%)	5 (2.1%)	
HbA1c testing and lab values			
HbA1c test (any) ^a^ post-baseline	219 (91.6%)	209 (89.3%)	*χ*^2^ = 0.74, *p* = 0.391
3+ HbA1c tests (post BL-12) ^a^	131 (54.8%)	105 (44.9%)	*χ*^2^ = 4.67, *p* = 0.031
Has HbA1c claim with value (BL) ^a^	157 (65.7%)	141 (60.3%)	*χ*^2^ = 1.50, *p* = 0.221
Has HbA1c claim with value (6–12) ^a^	94 (39.3%)	69 (29.5%)	*χ*^2^ = 5.07, *p* = 0.025
HbA1c value (6–12)	M = 8.21% (SD = 1.84%)	M = 8.16% (SD = 1.74%)	*t* = 1.15, *p* = 0.252
HbA1c < 7.0% (6–12)	29 (30.9%)	23 (33.3%)	*χ*^2^ = 0.11, *p* = 0.733
Has HbA1c values at BL AND 6–12	76 (31.8%)	60 (25.6%)	*χ*^2^ = 2.19, *p* = 0.140
HbA1c value change BL to 12	M = −0.26% (SD = 1.74%)	M = −0.51% (SD = 1.37%)	*t* = 1.03, *p* = 0.302
HbA1c value change BL to 12 ≥ 0.5%	22 (28.9%)	26 (43.3%)	*χ*^2^ = 1.94, *p* = 0.164
Recommended health screenings			
Retinal eye exam ^b^	117 (49.0%)	108 (46.2%)	*χ*^2^ = 0.23, *p* = 0.634
Kidney function panel ^b^	130 (54.4%)	126 (55.0%)	*χ*^2^ < 0.01, *p* = 0.971
Lipid panel ^b^	61 (25.5%)	57 (24.4%)	*χ*^2^ = 0.04, *p* = 0.833
Diabetes Complications			
Uncontrolled diabetes outpatient	134 (56.1%)	138 (59.0%)	*χ*^2^ = 0.41, *p* = 0.522
Uncontrolled diabetes ED/inpatient	44 (18.4%)	38 (16.2%)	*χ*^2^ = 0.39, *p* = 0.533
DCSI total score	1.70 (1.78)	2.15 (1.99)	*z* = 2.46, *p* = 0.014
Retinopathy	50 (20.9%)	56 (23.9%)	*χ*^2^ = 0.75, *p* = 0.387
Nephropathy	47 (19.7%)	53 (22.6%)	*χ*^2^ = 0.76, *p* = 0.384
Peripheral neuropathy	88 (36.8%)	96 (41.0%)	*χ*^2^ = 1.12, *p* = 0.290
Cerebrovascular complications	19 (7.9%)	29 (12.4%)	*χ*^2^ = 2.72, *p* = 0.099
Cardiovascular complications	55 (23.0%)	75 (32.1%)	*χ*^2^ = 5.27, *p* = 0.022
Peripheral vascular disease	40 (16.7%)	40 (17.1%)	*χ*^2^ = 0.03, *p* = 0.866
Metabolic complications	15 (6.3%)	13 (5.6%)	*χ*^2^ = 0.09, *p* = 0.767
Medication adherence			
No pharmacy claims	0	5 (2.1%)	
Unique medications	18.04 (8.33)	19.39 (8.65)	*t* = 1.75, *p* = 0.080
Statin coverage ≥ 80% of year ^a^	77 (32.2%)	98 (41.9%)	*χ*^2^ = 0.79, *p* = 0.375
Antidiabetic coverage ≥ 80% of year ^a^	131 (54.8%)	109 (46.6%)	*χ*^2^ = 0.36, *p* = 0.551
Healthcare Utilization			
Wellness visit (any)	78 (32.6%)	65 (27.8%)	*χ*^2^ = 1.32, *p* = 0.250
ED utilization (any)	139 (58.2%)	145 (62.0%)	*χ*^2^ = 0.71, *p* = 0.398
High ED utilization (>4/year)	32 (13.4%)	40 (17.1%)	*χ*^2^ = 1.26, *p* = 0.262
Hospitalization (any)	48 (20.1%)	58 (24.8%)	*χ*^2^ = 1.50, *p* = 0.220
90-day rehospitalization	15 (6.3%)	19 (8.1%)	*χ*^2^ = 0.60, *p* = 0.438

^a^ Only includes those with 1 or more claims for the medication or test. ^b^ Source: HEDIS Diabetes Care guidelines, 2022–2023. Note. Statistical comparisons were not made when cell sizes *n* < 5. Bolded text *p* < 0.05. Legend. *n* = sample size, M = mean, SD = standard deviation, *χ*^2^ = chi-squared test coefficient, *t* = *t*-test coefficient, HbA1c = glycated hemoglobin, BL = baseline, ED = emergency department, DCSI = Diabetes Complications Severity Index.

**Table 3:** Survey responses at 12 months follow-up by study group.

Survey Measures	Total *n* = 325	Usual Care *n* = 156	Navigation *n* = 169	Group Difference
Psychosocial factors	*n* (%) or M (SD)	*n* (%) or M (SD)	*n* (%) or M (SD)	*t* or *χ*^2^
Quality of Life SF-12v2 (T-scores)				
Aggregate physical health	36.28 (11.60)	36.02 (11.77)	36.55 (11.46)	*t* = 0.40, *p* = 0.713
Physical functioning	37.79 (12.52)	37.5 (12.97)	38.09 (12.07)	*t* = 0.41, *p* = 0.737
Role: physical	38.48 (10.47)	38.11 (10.55)	38.87 (10.40)	*t* = 0.64, *p* = 0.475
Bodily pain	36.17 (14.33)	35.52 (14.33)	36.86 (14.34)	*t* = 0.82, *p* = 0.412
General health	34.86 (10.92)	34.73 (10.47)	35 (11.40)	*t* = 0.22, *p* = 0.869
Aggregate mental health	45.24 (8.49)	45.19 (8.01)	45.28 (8.99)	*t* = 0.09, *p* = 0.862
Social functioning	40.95 (12.92)	41.71 (13.19)	40.14 (12.63)	*t* = 1.06, *p* = 0.329
Role: emotional	40.21 (12.68)	39.41 (12.62)	41.04 (12.72)	*t* = 1.14, *p* = 0.278
Vitality	49.44 (11.58)	49.21 (12.13)	49.68 (11.01)	*t* = 0.36, *p* = 0.718
Mental health	42.3 (7.94)	42.3 (7.39)	42.3 (8.51)	*t* < 0.01, *p* = 0.994
Perceived Stress Scale (Range: 0–16)	6.66 (3.29)	6.59 (3.33)	6.73 (3.26)	*t* = 0.35, *p* = 0.726
Diabetes Distress Scale (Range: 2–12)	4.96 (2.76)	4.86 (2.81)	5.07 (2.72)	*t* = 0.65, *p* = 0.515
PHQ Depression Symptoms (Range: 0–6)	1.86 (1.74)	1.89 (1.78)	1.83 (1.71)	*t* = 0.31, *p* = 0.756
Diabetes Self-Efficacy (Range: 0–10)	7.25 (1.81)	7.18 (1.81)	7.32 (1.81)	*t* = 0.69, *p* = 0.492
Social Support (Range: 0–16)	2.77 (1.13)	2.74 (1.09)	2.81 (1.16)	*t* = 0.58, *p* = 0.563
Sleep quality in past month				
Hours of sleep (Range: 0–10.5)	5.62 (2.34)	5.51 (2.41)	5.75 (2.26)	*t* = 0.90, *p* = 0.369
Quality of sleep (Range: 1–4)	1.61 (0.96)	1.56 (0.98)	1.66 (0.94)	*t* = 0.97, *p* = 0.332
Trouble sleeping (Range: 1–4)	2.02 (1.18)	1.99 (1.17)	2.04 (1.19)	*t* = 0.34, *p* = 0.733
Health behavior factors				
Diabetes self-management (Range 0–70)	42 (13.40)	42.11 (13.03)	41.88 (13.82)	*t* = 0.15, *p* = 0.883
General eating plan (Range: 0–7)	4.39 (2.15)	4.45 (2.10)	4.32 (2.20)	*t* = 0.50, *p* = 0.615
Diabetes-specific diet	4.25 (1.68)	4.24 (1.67)	4.25 (1.69)	*t* = 0.04, *p* = 0.967
Exercise	3.09 (2.25)	3.11 (2.34)	3.08 (2.17)	*t* = 0.13, *p* = 0.897
Glucose testing	4.47 (2.68)	4.67 (2.57)	4.27 (2.77)	*t* = 1.32, *p* = 0.188
Footcare	4.88 (2.24)	4.77 (2.22)	5.00 (2.26)	*t* = 0.91, *p* = 0.362
Had an HbA1c test in the previous 3 months (self-report)	229 (70.5%)	122 (76.3%)	107 (70.4%)	*χ*^2^ = 1.37, *p* = 0.242
Most recent HbA1c test result recall				*χ*^2^ = 4.04, *p* = 0.401
<7.0%	39 (17.0%)	24 (19.7%)	15 (14.0%)	
7.0–7.9%	42 (18.3%)	25 (20.5%)	17 (15.9%)	
8.0–8.9%	31 (13.5%)	18 (14.8%)	13 (12.1%)	
9.0%+	55 (24.0%)	27 (22.1%)	28 (26.2%)	
Unsure/Do not know	62 (27.1%)	28 (23.0%)	34 (31.8%)	
Social needs sum	1.73 (1.72)	1.63 (1.71)	1.84 (1.74)	*t* = 1.05, *p* = 0.297
Food	11 (3.6%)	3 (1.9%)	8 (5.3%)	
Transportation	28 (9.1%)	14 (8.9%)	14 (9.3%)	*χ*^2^ = 0.02, *p* = 0.885
Place to stay	12 (3.9%)	5 (3.2%)	7 (4.7%)	*χ*^2^ = 0.46, *p* = 0.496
Living space	49 (15.9%)	29 (18.4%)	20 (13.3%)	*χ*^2^ = 1.45, *p* = 0.229
Neighborhood safety	44 (14.3%)	27 (17.1%)	17 (11.3%)	*χ*^2^ = 2.08, *p* = 0.149
Utilities	52 (16.9%)	20 (12.7%)	32 (21.3%)	*χ*^2^ = 4.13, *p* = 0.042
Necessities	62 (20.1%)	27 (17.1%)	35 (23.3%)	*χ*^2^ = 1.87, *p* = 0.172
Unexpected expenses	162 (52.6%)	75 (47.5%)	87 (58.0%)	*χ*^2^ = 3.42, *p* = 0.064
Social isolation	41 (13.3%)	21 (13.3%)	20 (13.3%)	*χ*^2^ < 0.01, *p* = 0.991
Personal harm	33 (10.7%)	20 (12.7%)	13 (8.7%)	*χ*^2^ = 1.28, *p* = 0.258
Childcare	5 (1.6%)	3 (1.9%)	2 (1.3%)	
Other	41 (13.2%)	17 (10.6%)	24 (15.9%)	*χ*^2^ = 1.88, *p* = 0.170

Note. Percentages may not equal 100 due to rounding and missing data. Legend. *n* = sample size, M = mean, SD = standard deviation, *χ*^2^ = chi-squared test coefficient, *t* = *t*-test coefficient, SF = Short-Form Health Survey, PHQ = Patient Health Questionnaire, HbA1c = glycated hemoglobin.

## Data Availability

Survey data are available from authors upon reasonable request and IRB approval. Administrative and claims data are available from Louisiana Healthcare Connections with appropriate IRB approval and data sharing agreements.

## Supplementary Materials

The following supporting information can be downloaded at: https://www.mdpi.com/article/10.3390/ijerph21070936/s1, Supplementary Table S1. Procedure and drug codes used to identify outcomes from claims data. Supplementary Table S2. Care management outreach and engagement by study group (*n* = 473). Supplementary Table S3. Survey responses at 6 months follow-up by study group. Supplementary Table S4. Only significant time–group interactions of all comparisons explored over time.
