# Supplementary material for: A Randomized Controlled Trial Testing the Effects of a Social Needs Navigation Intervention on Health Outcomes and Healthcare Utilization among Medicaid Members with Type 2 Diabetes
Source: Int J Environ Res Public Health. 2024 Jul 18;21(7):936. doi: 10.3390/ijerph21070936 (PMC11277523; doi:10.3390/ijerph21070936)
Supplement: Supplementary file 1 [file ijerph-21-00936-s001.zip › ijerph-3073464-supplementary.pdf]

Supplement Table S1. Procedure and drug codes used to identify outcomes from claims data

Type of exam/use	CPT	CPT category II	HCSPCS codes
Statin medications[1]			
Anti-diabetic medications[1]			
HbA1c[2]	83036, 83037		
Retinal eye exam[2]	80061, 82465, 83700, 83701, 83704, 83718, 83721, 83722, 84478	3048F, 3049F, 3050F	
Kidney function panel[2]	98969, 98970, 98971, 98972, 99201, 99202, 99203, 99204, 99205, 99211, 99212, 99213, 99214, 99215, 99241, 99242, 99243, 99244, 99245, 99341, 99342, 99343, 99344, 99345, 99347, 99348, 99349, 99350, 99381, 99382, 99383, 99384, 99385, 99386, 99387, 99391, 99392, 99393, 99394, 99395, 99396, 99397, 99401, 99402, 99403, 99404, 99411, 99412, 99421, 99422, 99423, 99429, 99444, 99455, 99456, 99457, 99483	3074F, 3075F, 3077F, 3078F, 3079F, 3080F	G0071, G0402, G0438, G0439, G0463, G2010, G2012, G2061, G2062, G2063, T1015
Lipid panel[2]		3044F, 3046F, 3051F, 3052F	

References

1. Ryan JL, Franklin SM, Canterberry M, et al. Association of health-related social needs with quality and utilization outcomes in a Medicare advantage population with diabetes. *JAMA Network Open*. 2023;6(4):e239316.
2. National Committee for Quality Assurance (NCQA). HEDIS diabetes care guidelines, 2022-2023. Available from https://www.wellcarenc.com/content/dam/centene/wellcare/nc/pdfs/NC_Caid_Prov_HEDIS_Quick_Reference_Guide_2023_Eng_2023_R.pdf. Accessed 27 February 2024. 2023.

Supplement Table S2. Care management outreach and engagement by study group (*N*=473)

	Usual care (N=239)	Navigation (N=234)	
**Care management data:**	N (%)<br>M (SD), Range		χ^2, *p*
**Case type = social needs**	1 (0.4%)	205 (87.6%)	
Days until case was opened post-BL	97	15.41 (25.48), 0-169	
Days case was open/active	85.5	162.28 (33.62), 7-183	
**Notes files** (any study-related)	N/A	217 (92.7%)<br>4.36 (2.91), 1-20	
**Care plan (any)**	16 (6.7%)[a]	214 (91.5%)[b]	χ^2 = 399.17, *p* <.001
Problems (any)	11 (68.8%)<br>1.6 (1.7), 0-5[c]	214 (100%)<br>1.1 (0.5), 0-4[c]	χ^2 = 406.04, *p* <.001[c]
health-focused problems (any)	11 (68.8%)	58 (27.1%)	χ^2 = 67.52, *p* <.001[c]
social needs-focused problems (any)	4 (25.0%)	173 (80.8%)	
Goal (any)	13 (81.3%)<br>2.1 (2.3), 0-9[c]	214 (100%)<br>1.60 (1.1), 1-7[c]	χ^2 = 406.04, *p* <.001[c]
Intervention (any)	14 (87.5%)<br>6.6 (7.3), 0-28[c]	214 (100%)<br>2.8 (1.7), 0-15[c]	χ^2 = 402.59, *p* <.001[c]
Barrier (any)	12 (75.0%)<br>2.1 (2.3), 0-7[c]	84 (39.3%)<br>0.5 (0.7), 0-4[c]	χ^2 = 105.84, *p* <.00[illegible][c]

[a] Although there were individuals in the usual care group with care plans, none of them were managed by the two study navigators who conducted our intervention.

[b] Only care plans managed by a study navigator are included in this count, which excluded n=4 participants with care plans managed by other care managers.

[c] Descriptives and comparisons only include participants with a care plan

Statistical comparisons were not made when cell sizes n<5

Supplement Table S3. Survey responses at 6 month follow up by study group

Survey measures	Total N=325	Usual Care N=156	Navigation N=169	Group difference
**Psychosocial factors**	N% or M(SD)	N% or M(SD)	N% or M(SD)	*t* or χ^2
Quality of Life SF-12v2 (T-scores)				
Aggregate physical health	37.03 (11.85)	37.23 (11.74)	36.86 (11.97)	$t = 0.27$, $p = .787$
Physical functioning	38.35 (12.78)	38.71 (12.70)	38.04 (12.88)	$t = 0.46$, $p = .644$
Role: physical	39.24 (10.65)	39.91 (11.13)	38.64 (10.21)	$t = 1.05$, $p = .294$
Bodily pain	36.54 (14.03)	36.65 (14.41)	36.44 (13.72)	$t = 0.13$, $p = .894$
General health	35.07 (10.97)	35.73 (11.22)	34.48 (10.74)	$t = 1.01$, $p = .315$
Aggregate mental health	45.42 (8.04)	45.90 (7.93)	44.99 (8.13)	$t = 0.99$, $p = .324$
Social functioning	41.40 (13.19)	41.94 (13.28)	40.92 (13.13)	$t = 0.67$, $p = .501$
Role: emotional	39.75 (12.74)	40.48 (12.91)	39.10 (12.59)	$t = 0.96$, $p = .340$
Vitality	51.17 (11.53)	51.17 (11.57)	51.16 (11.52)	$t < .01$, $p = .995$
Mental health	42.48 (7.25)	42.71 (7.15)	42.26 (7.36)	$t = 0.54$, $p = .586$
Perceived Stress Scale (Range: 0-16)	6.44 (3.45)	6.47 (3.40)	6.40 (3.50)	$t = 0.18$, $p = .857$
Diabetes Distress Scale (Range: 2-12)	5.13 (2.77)	5.2 (2.84)	5.07 (2.71)	$t = 0.42$, $p = .676$
PHQ Depression Symptoms (Range: 0-6)	1.99 (1.79)	2.02 (1.82)	1.96 (1.77)	$t = 0.28$, $p = .783$
Diabetes Self-Efficacy (Range: 0-10)	7.26 (1.87)	7.23 (1.90)	7.29 (1.85)	$t = 0.26$, $p = .792$
Social Support (Range: 0-16)	2.74 (1.10)	2.70 (1.10)	2.77 (1.10)	$t = 0.61$, $p = .542$
Sleep quality in past month				
Hours of sleep (Range: 0-10.5)	5.69 (2.13)	5.53 (2.24)	5.84 (2.02)	$t = 1.29$, $p = .199$
Quality of sleep (Range: 1-4)	1.63 (1.03)	1.63 (1.05)	1.63 (1.01)	$t = 0.04$, $p = .965$
Trouble sleeping (Range: 1-4)	1.89 (1.20)	1.86 (1.20)	1.91 (1.20)	$t = 0.39$, $p = .695$
**Health behavior factors**				
Diabetes self-management (Range 0-70)	41.18 (13.77)	41.21 (13.87)	41.14 (13.72)	$t = 0.04$, $p = .963$
General eating plan (Range: 0-7)	4.35 (2.19)	4.47 (2.16)	4.23 (2.21)	$t = 0.97$, $p = .333$
Diabetes-specific diet	4.27 (1.68)	4.16 (1.80)	4.38 (1.56)	$t = 1.22$, $p = .223$
Exercise	2.98 (2.17)	2.99 (2.20)	2.96 (2.14)	$t = 0.11$, $p = .914$
Glucose testing	4.60 (2.66)	4.69 (2.63)	4.51 (2.69)	$t = 0.62$, $p = .535$
Footcare	4.48 (2.46)	4.41 (2.55)	4.55 (2.39)	$t = 0.51$, $p = .609$
Most recent A1c test result recall				$\chi^2 = 9.21$, $p = .084$
< 7.0%	37 (14.7%)	23 (18.5%)	14 (11.0%)	
7.0-7.9%	50 (19.9%)	19 (15.3%)	31 (24.4%)	
8.0-8.9%	39 (15.5%)	24 (19.4%)	15 (11.8%)	
9.0%+	47 (18.7%)	20 (16.1%)	27 (21.3%)	
Unsure/Don't know	78 (31.1%)	38 (30.6%)	40 (31.5%)	
**Social needs sum**	1.92 (1.79)	1.78 (1.73)	2.05 (1.83)	$t = 1.37$, $p = .173$
Food	17 (4.8%)	9 (5.2%)	8 (4.4%)	$\chi^2 = 0.13$, $p = .722$
Transportation	36 (10.1%)	15 (8.6%)	21 (11.5%)	$\chi^2 = 0.80$, $p = .371$
Place to stay	10 (2.8%)	4 (2.3%)	6 (3.3%)	
Living space	51 (14.3%)	20 (11.5%)	31 (16.9%)	$\chi^2 = 2.16$, $p = .142$
Neighborhood safety	45 (12.6%)	22 (12.6%)	23 (12.6%)	$\chi^2 < .01$, $p = .983$
Utilities	61 (17.1%)	25 (14.4%)	36 (19.7%)	$\chi^2 = 1.77$, $p = .183$
Necessities	65 (18.2%)	25 (14.4%)	40 (21.9%)	$\chi^2 = 3.36$, $p = .067$
Unexpected expenses	164 (45.9%)	75 (43.1%)	89 (48.6%)	$\chi^2 = 1.10$, $p = .295$
Social isolation	49 (13.7%)	25 (14.4%)	24 (13.1%)	$\chi^2 = 0.12$, $p = .731$
Personal harm	23 (6.4%)	14 (8%)	9 (4.9%)	$\chi^2 = 1.45$, $p = .229$
Childcare	5 (1.4%)	3 (1.7%)	2 (1.1%)	
Other	53 (16.6%)	22 (14.4%)	31 (18.6%)	$\chi^2 = 1.01$, $p = .303$

Note. Percentages may not equal 100 due to rounding and missing data. Legend. N=sample size, M=mean, SD=standard deviation, χ^2=chi-squared test coefficient, *t*=t-test coefficient, SF=Short Form Health Survey, PHQ=Patient Health Questionnaire, HbA1c=glycated hemoglobin

Supplement Table S4. Only significant time*group interactions of all comparisons explored over time

Quality of Life SF-12v2		*β* (95% CI)	*t, p*
**Physical health (aggregate)**	Time	0.07 [-0.03, 0.16]	*t* = 1.38, *p* = .169
	Study group	-0.13 [-0.28, 0.02]	*t* = -1.75, *p* = .082
	Time * study group	0.15 [-0.02, 0.32]	*t* = 1.77, *p* = .078
Physical functioning	Time	0.10 [0.01, 0.19]	*t* = 2.19, *p* = .029
	Study group	-0.08 [-0.23, 0.07]	*t* = -100, *p* = .317
	Time * study group	0.08 [-0.07, 0.23]	*t* = 1.10, *p* = .271
Role: physical	Time	0.10 [0.01, 0.19]	*t* = 2.20, *p* = .029
	Study group	-0.09 [-0.28, 0.09]	*t* = -0.99, *p* = .324
	Time * study group	0.08 [-0.08, 0.24]	*t* = 0.96, *p* = .339
Bodily pain	Time	< .01 [-0.08, 0.10]	*t* = 0.17, *p* = .868
	Study group	-0.11 [-0.27, 0.05]	*t* = -1.31, *p* = .193
	Time * study group	0.12 [-0.03, 0.27]	*t* = 1.54, *p* = .125
General health	Time	0.17 [0.09, 0.26]	*t* = 3.97, *p* < .001
	Study group	-0.06 [-0.23, 0.10]	*t* = -0.74, *p* = .461
	Time * study group	0.03 [-0.11, 0.18]	*t* = 0.46, *p* = .649
**Mental health (aggregate)**	Time	0.06 [-0.04, 0.15]	*t* = 1.17, *p* = .241
	Study group	0.06 [-0.17, 0.29]	*t* = 0.51, *p* = .614
	Time * study group	-0.05 [-0.22, 0.11]	*t* = -0.63, *p* = .528
Social functioning	Time	0.16 [0.07, 0.25]	*t* = 3.59, *p* < .001
	Study group	0.11 [-0.05, 0.28]	*t* = 1.37, *p* = .172
	Time * study group	-0.15 [-0.30, -0.01]	*t* = -2.07, *p* = .039
Role: emotional	Time	0.08 [-0.02, 0.17]	*t* = 1.59, *p* = .114
	Study group	-0.10 [-0.28, 0.08]	*t* = -1.11, *p* = .267
	Time * study group	0.10 [-0.06, 0.27]	*t* = 1.25, *p* = .213
Vitality	Time	-0.17 [-0.26, -0.08]	*t* = -3.61, *p* < .001
	Study group	-0.15 [-0.32, 0.03]	*t* = -1.63, *p* = .105
	Time * study group	0.13 [-0.02, 0.29]	*t* = 1.67, *p* = .095
Mental health	Time	0.04 [-0.05, 0.13]	*t* = 0.87, *p* = .386
	Study group	0.01 [-0.41, 0.43]	*t* = 0.05, *p* = .963
	Time * study group	< .01 [-0.15, 0.15]	*t* = 0.03, *p* = .975
**Diabetes self-care activities (aggregate)**	Time	0.12 [0.04, 0.20]	*t* = 2.98, *p* = .003
	Study group	0.04 [-0.09, 0.18]	*t* = 0.61, *p* = .543
	Time * study group	-0.07 [-0.21, 0.07]	*t* = -0.96, *p* = .339
Followed daily vs. less often		*OR* (95% CI)	*z, p*
General diet	Time	1.98 [1.19, 3.30]	*z* = 2.62, *p* = .009
	Study group	1.80 [0.59, 5.49]	*z* = 1.03, *p* = .302
	Time * study group	0.87 [0.62, 1.21]	*z* = -0.84, *p* = .404
Diabetes-specific diet	Time	0.86 [0.40, 1.85]	*z* = -0.38, *p* = .702
	Study group	0.75 [0.08, 7.20]	*z* = -0.25, *p* = .802
	Time * study group	0.99 [0.40, 2.45]	*z* = -0.01, *p* = .990

Exercise	Time	1.60 [0.80, 3.21]	z = 1.32, p = .187
	Study group	1.95 [0.27, 14.04]	z = 0.66, p = .507
	Time * study group	0.77 [0.38, 1.56]	z = -0.73, p = .464
Tested blood glucose	Time	1.83 [0.93, 3.59]	z = 1.76, p = .078
	Study group	0.95 [0.81, 1.13]	z = -0.55, p = .586
	Time * study group	0.79 [0.62, 0.99]	z = -1.98, p = .047
Checked feet	Time	1.14 [0.92, 1.43]	z = 1.19, p = .236
	Study group	0.93 [0.48, 1.80]	z = -0.22, p = .827
	Time * study group	1.08 [0.83, 1.42]	z = 0.59, p = .554

Note. Quality of Life Time: 1= Baseline, 2 = 6 months post-baseline, 3 = 12 months post-baseline; Diabetes Self-Care Activities Time: Time: 1= Baseline, 2 = 3 months post-baseline, 3 = 6 months post-baseline, 4 = 12 months post-baseline; Study group: 1 = Control, 2 = Navigation
